# Pervaporation Separation of Isopropanol/Water Using Zeolite Nanosheets: A Molecular Simulation Study

**DOI:** 10.1021/acs.jpcb.4c04237

**Published:** 2024-08-26

**Authors:** Ming-Yen Tsai, Li-Chiang Lin

**Affiliations:** † Department of Chemical Engineering, National Taiwan University, No. 1, Sec. 4, Roosevelt Road, Taipei 10617, Taiwan; ‡ William G. Lowrie Department of Chemical and Biomolecular Engineering, The Ohio State University, 151 W. Woodruff Avenue, Columbus, Ohio 43210, United States

## Abstract

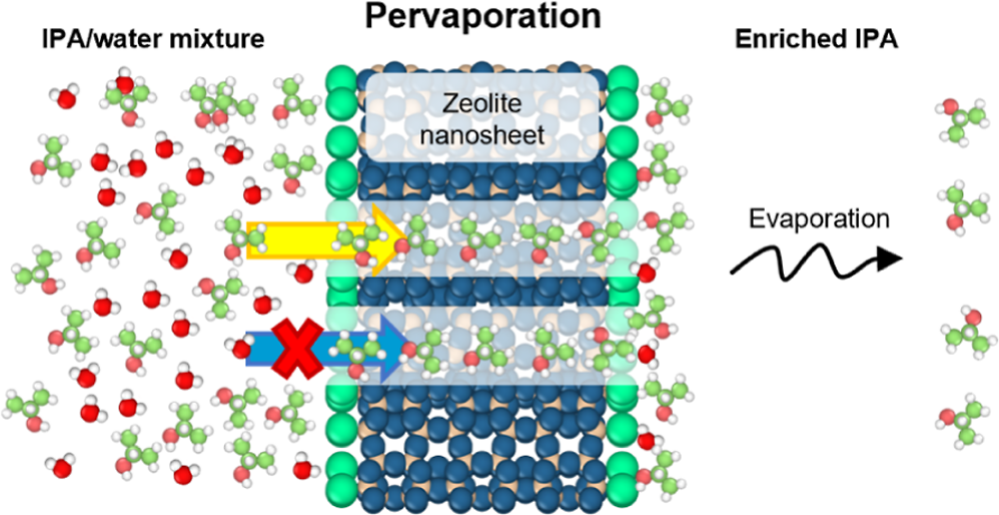

Reducing greenhouse gas emissions plays a crucial role in slowing down the rise of the global temperature. One of the viable options is to employ renewable energy sources such as alcohols that can be produced from biomass. Specifically, one of the most common alcohols is isopropanol (IPA). Energy-intensive distillation processes are however involved in its production because of the rather low product concentration from fermentation. Membrane technologies, specifically pervaporation (PV), represent a promising alternative to the IPA/water separation. Particularly, employing zeolite nanosheets as PV membranes may provide great opportunities to extract IPA owing to their ultrathin and hydrophobic nature. By employing molecular dynamics simulations, this study conducts a systematic study on a diverse set of nanosheet candidates with the aim of exploring their potential and identifying top-performing structures. The best candidate among structures studied herein is predicted to offer an exceptional IPA/water selectivity of more than 400 with an unprecedentedly large flux. Structure–property–performance relationships have also been established to offer insights into the rational design of PV membranes with improved performance.

## Introduction

Reducing greenhouse gas emissions is crucial to mitigating the escalated global temperature.^1,2^ Systematic and coordinated efforts are urgently needed to reduce the release of carbon dioxide into the atmosphere, achieving net-zero carbon dioxide emissions.^3−6^ One of the viable strategies involves the utilization of renewable energies.^7,8^ Specifically, renewable energy sources derived from biomass fermentation represent a strategically important direction. This involves the conversion of biobased materials into substances with a high energy density, such as methanol, ethanol, and different types of alcohol.^9−11^ Isopropanol (IPA) is one of the most common energy sources of alcohols and can also be used for a wide range of applications including rubbing alcohol, cleaners, and cosmetics.^12,13^

Fermentation approaches employed to yield high energy-density substances usually encounter challenges associated with low-concentration alcohol products (e.g., less than 5 wt % for IPA).^14−16^ This as a consequence necessitates further separation processes to extract anhydrous alcohols. Conventionally, distillation has been widely used to separate IPA from its dilute aqueous mixture.^17,18^ However, distillation is notoriously known for its energy-intensive nature, high capital and operating costs, and the formation of azeotropic mixtures (i.e., an azeotropic point of 87.4 wt % for the IPA/water mixture).^19^ There is therefore a need to develop alternative processes to the traditional distillation methods.

In recent decades, membrane technologies, particularly pervaporation (PV), have drawn substantial attention as a more energy-efficient alternative to distillation.^20,21^ Experimental studies reported in the literature to date have primarily focused on pervaporation membranes comprising polymers including poly(vinyl alcohol), cellulose, etc. Polymeric membranes typically exhibit hydrophilic characteristics and result in the so-called dehydrating process (i.e., preferentially allowing the permeation of the major component–water).^22^ As an example, Van Baelen et al. reported that Pervap 2201 can successfully separate an 85 wt % IPA/water solution with a flux of ∼220 (g/m^2^ h) and a water-to-IPA separation factor of ∼400.^23^ Smuleac et al. also demonstrated that CMS-3 can effectively separate a 98.7 wt % IPA/water solution, offering a flux of ∼50 (g/m^2^ h) and a water-to-IPA separation factor of ∼500.^24^ Polymeric membranes are, however, prone to swelling. While the swollen membranes may lead to an enhanced flux, a compromised separation factor also occurs. More importantly, such a dehydration process with polymeric membranes may in fact not be preferred when dealing with a rather low-concentration alcohol solution. Investigations have thus been conducted into the mixed matrix membranes (MMMs) with hydrophobic inorganic zeolite materials such as zeolites. For instance, Kamelian et al.^25^ developed a MMM membrane consisting of silicalite-1 and polydimethylsiloxane (PDMS) and achieved an alcohol-to-water separation factor of 17.24 and a total flux of 3.64 kg m^–2^ h^–1^. However, the performance of MMMs is still constrained by the polymer material. To this end, membranes comprising of pure nanoporous materials such as the aforementioned zeolites and metal–organic frameworks, leveraging their versatile surface chemistry and mechanical strengths, become of particular interest.^26−28^ Specifically, the former has been quite extensively studied as pervaporation membranes.^29,30^ Shu et al. have shown that pure-silica MFI zeolite (silicalite-1) with a thickness of approximately 3 μm, thanks to their hydrophilic nature, can achieve a superior ethanol-to-water separation factor of nearly 50.^31^ Such membranes can extract the minor component (i.e., alcohol) from its rather dilute solution, leading to reduced energy consumptions.

While a high separation factor is crucial, it is also important to achieve high flux for the large-scale deployment of the PV process. This dual requirement ensures not only an effective but also efficient separation. Zeolite nanosheets,^32,33^ a special class of zeolite materials synthesized in a nanoscale thickness capable of offering an ultrashort diffusion path may exhibit remarkable potential.^34^ For example, Jeon et al. proposed a synthesis method that produces MFI-type zeolite membranes with a thickness of approximately 5 nm.^35^ These zeolite nanosheets form thin, defect-free coatings that effectively cover porous substrates, leading to high *p*-xylene permeance and excellent *p*-xylene-to-*o*-xylene separation factors when separating *p*-xylene and *o*-xylene mixtures. Furthermore, zeolite nanosheets exhibit excellent thermal stability and mechanical strength, making them well-suited for separation processes.^36−38^ To date, a handful of zeolites included in the international zeolite association database have been synthesized as nanosheets. Moreover, more than 800,000 2D zeolite nanosheets have been computationally predicted.^39,40^ Such a large material space should open up tremendous opportunities for breakthroughs in IPA/water PV separation.

In this study, state-of-the-art molecular dynamics (MD) simulations are employed to explore the potential of zeolite nanosheets in IPA/water separation and to identify promising candidates. It should be noted that only siliceous zeolites are considered in this work. Those containing aluminum (i.e., aluminosilicate zeolites) may become too hydrophilic and thus have a relatively weaker affinity toward IPA, potentially resulting in a poor IPA-to-water separation performance. A comprehensive analysis to reveal factors, such as adsorption capability and surface properties, dominating their PV performance is also conducted. Besides, the structure–property–performance relationship of zeolite nanosheets is established to guide the future rational design of better PV membranes.

## Computational Details

A diverse set of zeolite structures are investigated as PV membranes in this study for IPA/water separation. This includes MFI,^32,34,41−44^ FER,^45^ BEC,^46^ and MRE^47^ that have been experimentally synthesized. It should be noted that MFI, FER, and BEC are also studied with different surface preparations (i.e., termination and orientation) to explore their effects. Specifically, two MFI-based structures (i.e., termed MFI and MFI_zigzag) are created by cutting the MFI structure at fraction coordinates of 0 and 0.25, respectively along the crystallographic *a*-direction. Two FER-based structures (i.e., designated as FERr and FER) that exhibit opposite surfaces interfacing with the feed-side and the permeate-side solutions are also considered. Moreover, two BEC-based structures (i.e., denoted as BECa and BECc), with permeation flow respectively along the crystallographic *a*- and *c*-directions, are studied. Besides, OSI, ATS, IWV, AET, ETR, and IRR are included for their simple 1D channel structures with varying pore apertures. For all the studied candidates, their surface dangling bonds are saturated with silanol groups. In total, as summarized in Table S1, 14 zeolite candidates with a wide spectrum of the pore limiting diameter (PLD, calculated by Zeo++ software^48,49^), ranging from 4.29 to 11.71 Å, are studied herein.

**Figure 1:**
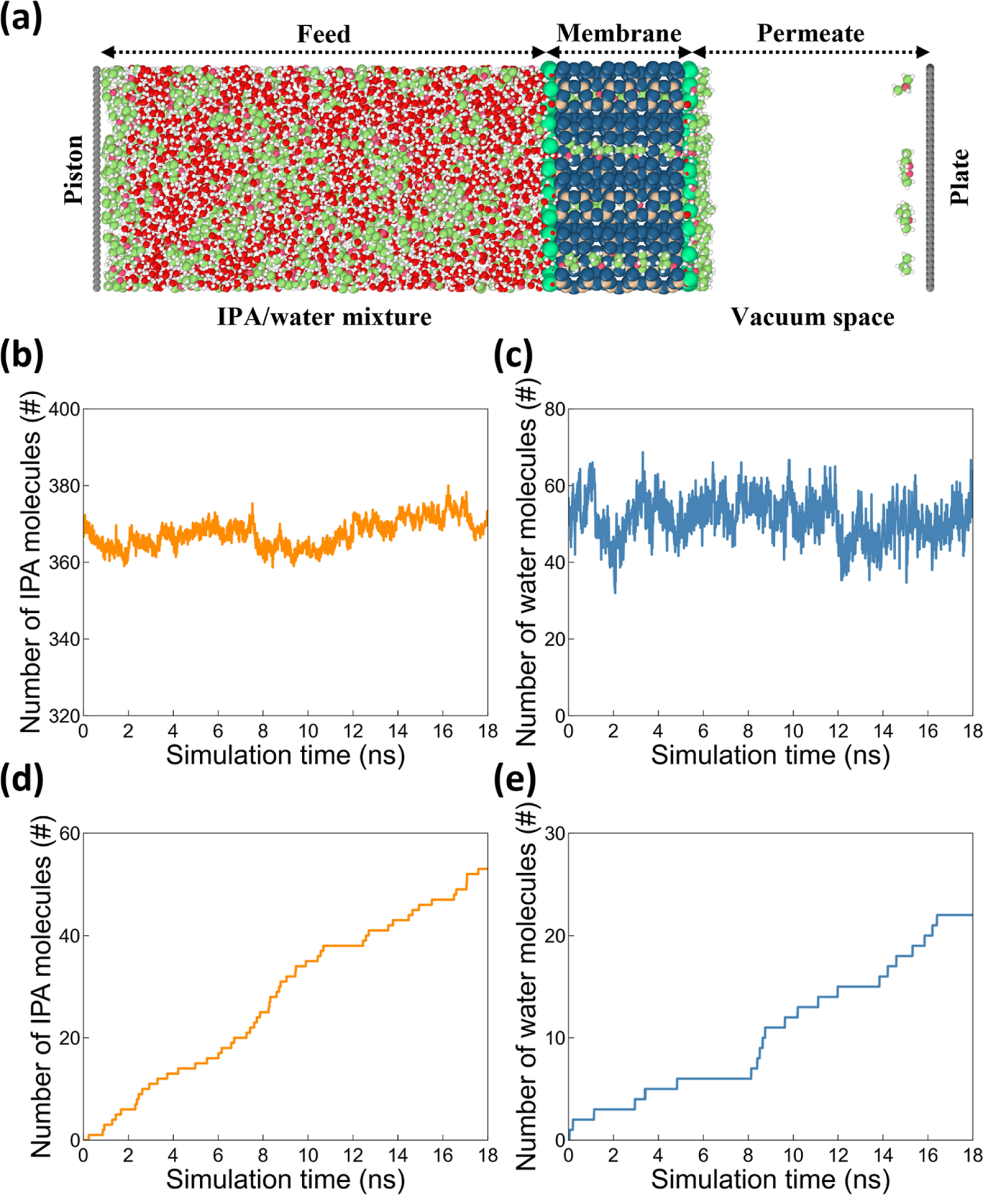
(a) Illustration of the simulation domain featuring the zeolite nanosheet as the active membrane layer. (b–e) typical outcomes of the simulations: the number of (b, d) IPA and (c, e) water molecules residing (b, c) in the membrane and (d, e) on the adsorbing plate as the function of simulation time.

To probe their separation performance in the extraction of IPA from IPA/water mixtures, MD simulations, implemented in the open-source LAMMPS package,^50^ are carried out. The simulation system is designed to have a sandwich-like configuration, as widely adopted in studies reported in the literature,^51−57^ to directly mimic a PV separation process. The system comprises, from the left to the right, the feed side (i.e., 40 wt % IPA/water mixture), the active membrane layer (i.e., zeolite nanosheet), and the permeate side (i.e., vacuum) as depicted in Figure 1 (a). Two rigid graphene layers are also included. The one on the feed side acts as a piston to maintain the pressure of the feed mixture to be at 1 atm, while the one placed on the permeate side serves as an adsorbing plate to capture all permeated molecules in order to maintain the vacuum condition.

These simulations are conducted in the canonical ensemble (*NVT* ensemble) with the system temperature modulated at 333 K, a commonly adopted operation temperature for alcohol/water pervaporation processes,^23,58,59^ using the Nosé–Hoover thermostat and a damping factor of 100 time steps (i.e., 100 fs).^60^ For describing nonbonded intermolecular interactions, both 12–6 Lennard-Jones (L-J) potential with a cutoff radius of 12 Å and the long-range electrostatic interactions computed using the particle–particle particle-mesh method with a precision of 10^–6^ are used. Nanosheet membranes are modeled using the potential developed by Emami et al.^61^ The force fields describing IPA and water are adopted from the OPLS-AA^62^ and the TIP4*P*/2005 model,^63^ respectively. Besides, the Lennard-Jones parameters for the carbon atoms of both rigid graphene pistons are taken from the OPLS-AA force field. The dimensions of the simulation domain are approximately 90 × 90 × 220 Å, and the active layer has a thickness of ∼24 Å. For each studied nanosheet PV membrane, an equilibration step of at least 20 ns under the *NVT* ensemble is first conducted to saturate the membrane with the feed-side mixture. This step ensures the number of molecules for both IPA and water inside the membranes remains nearly constant as depicted in Figure 1(b and c). The separation performance of the studied membrane is then assessed per trajectories collected from another production run of 12 ns. Specifically, two key performance metrics, i.e., separation factor (α) and flux (*J*), are calculated from the observed changes in molecular numbers collected on the adsorbing plate, as shown in Figure 1(d–e), per the following eqs 1 and 2.
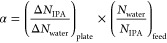
1
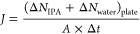
2where Δ*N*_IPA,plate_ and Δ*N*_water,plate_ represent the change in the number of IPA and water molecules, respectively, on the adsorbing plate over a time interval Δ*t*, *A* represents the area of the membrane that is parallel to the permeation direction, and *N*_IPA,feed_ and *N*_water,feed_ are respectively the number of IPA and water molecules located in the feed-side region at the beginning of the performance sampling. It should be noted that the convergence of each PV simulation are determined by two key indicators. First, as shown in Figure 1(b and c), the membrane should be saturated with both IPA and water molecules; their molecular number should remain at approximately constants. Second, as shown in Figure 1(d and e), a steady-state flow should be observed (i.e., linear increase in the number of molecules on the adsorbing plate over time).

To better understand the permeation mechanism and factors controlling the separation performance, both the density and the Helmholtz free energy profiles of IPA and water along the permeation direction are also determined per the MD trajectories. Specifically, the positions of IPA and water molecules, determined by the middle carbon and oxygen atoms, respectively, are first projected onto the permeation direction (*z*-axis) to yield the corresponding concentration profiles along the permeation channel. Subsequently, the free energy profile of IPA and water can be derived, as reported in studies such as that by Wang et al.,^51^ per the equation shown below
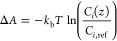
3where *k*_b_ is the Boltzmann constant, *T* is the temperature, *C*_*i*_ is the concentration of species *i* as a function of the *z*-coordinate, and *C*_*i*,ref_ represents the reference concentration that is chosen as the maximum concentration of the species *i*. This equation is originated from the fact that the simulation system is in an *NVT* ensemble. Despite that PV simulation is not in a true equilibrium condition, the observed probability of finding molecules should still be approximately related to the Helmholtz free energy per the definition of the ensemble. Though, it should be noted that, owing to the fact that there can be only few water molecules present in highly IPA-selective zeolites, sampling uncertainties in the free energy profile of water can be quite large. A more effective approach may be to use umbrella sampling combined with the weighted histogram analysis method.^64,65^

## Results and Discussion

This section first summarizes the MD-predicted performance of 14 studied nanosheet candidates, followed by discussing key factors controlling the PV separation performance as well as shedding light on the correlation between their geometric features and separation performance.

### PV Performance of Zeolite Nanosheets for IPA/Water Separation

**Figure 2:**
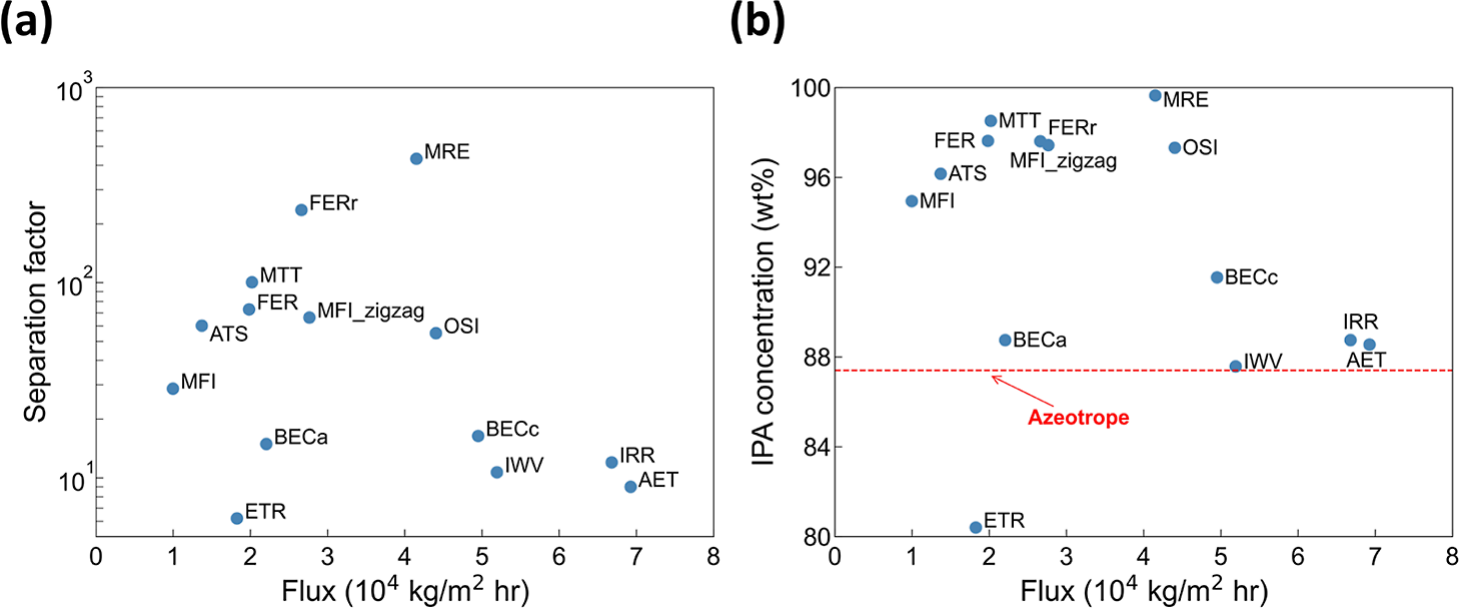
MD-predicted (a) separation factor and (b) corresponding IPA concentration as a function of flux for all studied zeolite nanosheets. Tabulated data can be found in Table S2. In (b), the red line indicates the azeotropic composition of the IPA/water solution (i.e., 87.4 wt % IPA).

Figure 2(a) shows the MD-predicted PV separation performance of all studied nanosheet structures to separate a 40 wt % IPA/water mixture at 333 K. All zeolites demonstrate a high flux with a magnitude of 10^4^ kg/m^2^ h, due to their ultrathin nature. This is notably higher than polymeric membrane as summarized in Tables 1 and S2.^23,24,66,67^ Indeed, such two-dimensional nanosheets have demonstrated significant potential for separation applications due to their exceptional molecular transport properties, as observed in experiments.^68,69^ Moreover, distinct from hydrophilic polymeric systems that preferentially allow the permeation of water, zeolite nanosheets studied herein offer a selective permeation of IPA. The results also show that zeolite nanosheets, if meticulously selected, can offer an exceptionally large IPA-to-water separation factor of approximately 430 (i.e., zeolite MRE). A wide range of separation factors are however observed, with the lowest to be as small as approximately 6 (i.e., zeolite ETR). Nonetheless, Figure 2(b) shows that all studied nanosheets except the aforementioned ETR, can extract IPA with a concentration higher than the azeotropic composition by merely a single PV step. The best zeolite, MRE, can even reach a concentration of 99.7 wt % IPA from a 40 wt % feed-side solution. As noted above, while only a handful of nanosheets have been synthesized, over 800,000 possible nanosheet structures have been computationally predicted. To this end, it remains important to shed light on the dominant factors such as adsorption selectivity, pore sizes, surface properties, and other characteristics affecting the separation performance, especially the separation factor. These aspects will be discussed in detail next.

**Table 1:** Comparison of the IPA/Water PV Separation Performance Between Polymeric Membranes and the Best Candidate, Zeolite MRE, Identified in This Study

membrane	feed mixture (wt %)	temperature (K)	separation factor	flux (kg/m^2^ h)	process^a^	reference
pervap 2201	85	333	∼400	∼0.22	dehydration	(23)
perfluorpolymer coated on PAN (CMS-3)	98.7	298	500	0.05	dehydration	(24)
polybenzoxazinone (PBOZ)	90	333	5000	0.003	dehydration	(66)
chitosan modified polybenzimidazole (PBI)	70	343	∼115	∼0.25	dehydration	(67)
MRE nanosheet	40	333	431.92	41504.9	extraction	this work

a The separation factor shown in this table is defined per the process type; dehydration pertains to the water-to-IPA separation factor (water/IPA) and vice versa for the extraction process (IPA/water).

### Role of Adsorption Selectivity

**Figure 3:**
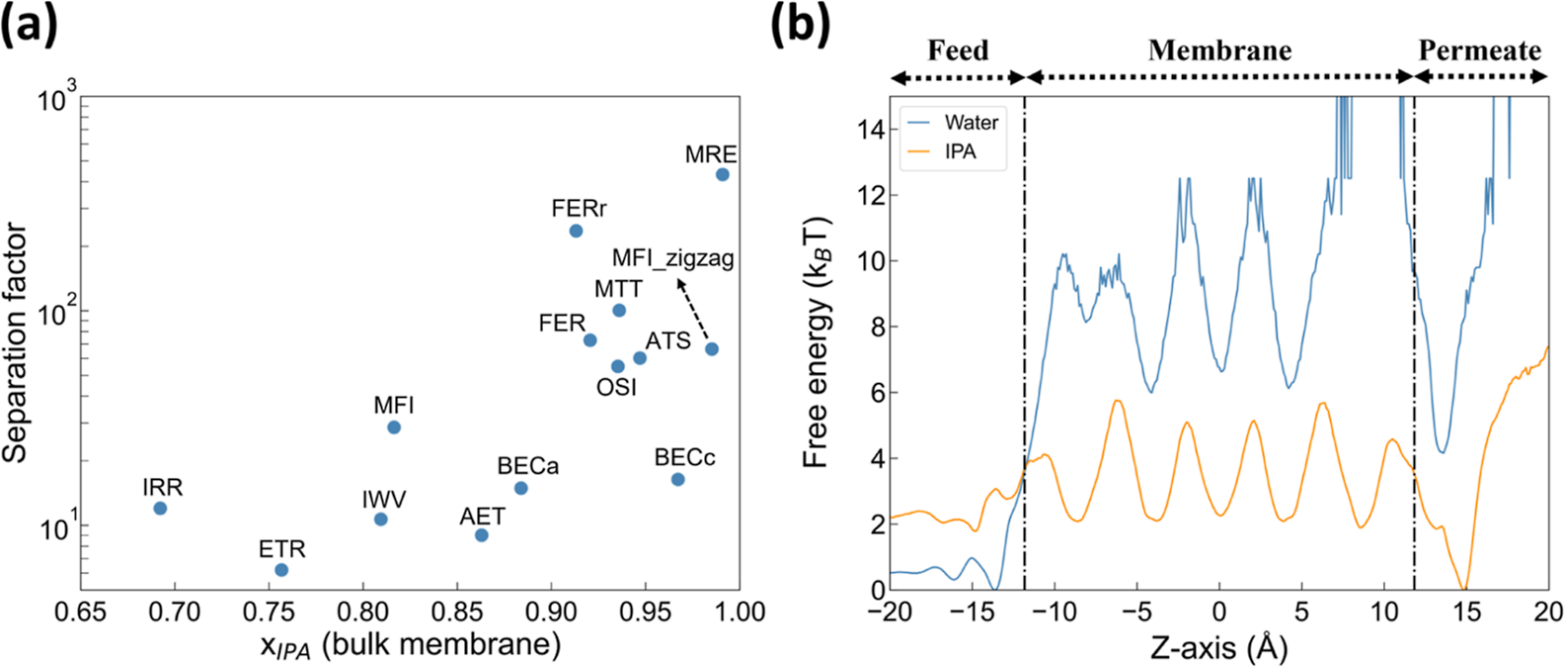
(a) Correlation between the PV separation factor and the IPA mole fraction (*x*_IPA_) in the bulk membrane. (b) The free energy profile of water and IPA in zeolite MRE with the black dotted lines represent the locations of external surfaces of the membrane.

Figure 3(a) illustrates that there exists a strong and positive correlation between the separation factor and the IPA mole fraction in the bulk membrane, suggesting that the observed separation performance is largely driven by the selective adsorption of IPA over water in the adopted membrane. We note that the mole fraction of IPA in the bulk membrane is calculated by analyzing the molecular trajectories collected from the MD simulations. The bulk membrane region is defined as depicted in Figure S1. When the IPA mole fraction in the bulk membrane is between 0.9 and 1.0, zeolites typically exhibit decent separation factors. This appears reasonable, provided that the membrane serves as a strong IPA adsorbent to selectively allow the entrance of IPA from the rather dilute feed solution. From the molecular trajectories as introduced in the Methods section above, the free energy profile of the most promising nanosheet – MRE – is also quantified and shown in Figure 3(b). As a reflection to the strong adsorption of IPA over water in the zeolite, such hydrophobic nature effectively imposes a large barrier of ∼10 *k*_B_*T* (∼6.6 kcal/mol) on water upon entering the zeolite membrane. Moreover, because the entire membrane is saturated with IPA, as shown in Figure S2, water molecules in the bulk membrane, though having a smaller size, experience a larger transport barrier, relative to IPA molecules, by ∼3 *k*_B_*T* (∼2.0 kcal/mol), which also contributes to the selective separation of IPA over water.

**Figure 4:**
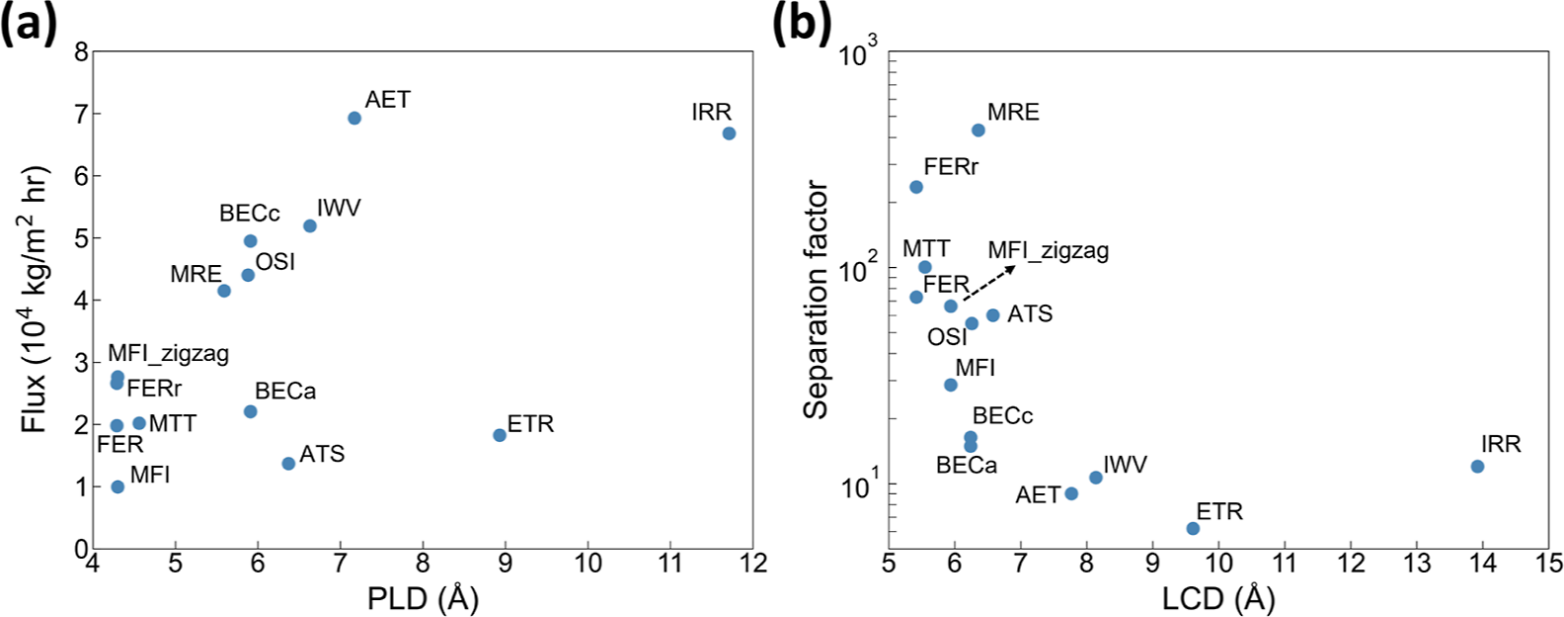
Correlations between (a) flux and PLD as well as (b) separation factor and LCD.

Provided that the adsorption and diffusion properties of porous materials have been generally considered to be a strong function of structure features,^70−74^ it would be advantageous if one could use some easy-to-compute geometric features to preliminarily probe the PV performance of nanosheet candidates. Figure 4(a) shows there exists a positive correlation between PLD and the flux. This observation should be deemed intuitive, given a larger permeation bottleneck straightforwardly permits faster permeation. However, outliers (i.e., ATS and ETR) do interestingly exist. ATS has a comparable pore size with BECc (i.e., PLD: 6.37 and 5.91 Å for ATS and BECc, respectively), but its flux is approximately 3.5 times lower than the latter. This lower flux can be attributed to the slow permeation arising from the topology of ATS, as will be detailed later. Similarly, ETR has a flux value that is well below expectations even when compared to zeolite membranes with smaller pore sizes (i.e., BECa and BECc). This discrepancy may be a result of its low channel density (0.27 nm^–2^), which is the smallest among all studied zeolite nanosheets (see Table S1). Figure 4(b) further demonstrates that zeolites with the largest cavity diameter (LCD) of approximately 6 Å likely to exhibit the highest separation selectivity. This observation is related to the confinement effect, given an LCD of 6 Å can be commensurate with the size of IPA (i.e., a kinetic diameter of 4.7)^75^ for favorable IPA-zeolite interactions. A similar observation has also been made in a previous study for ethanol/water separation.^53^

**Figure 5:**
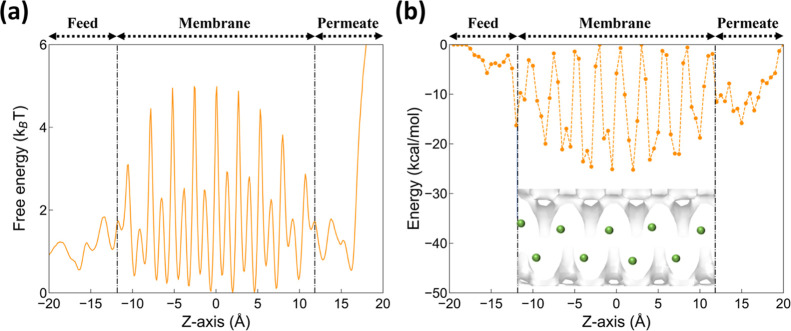
(a) Helmholtz free energy of IPA and (b) the IPA-membrane interaction energy in zeolite ATS along the permeation direction (i.e., *Z*-direction). The black dotted lines represent the locations of the ATS surfaces. The inset shown in (b) illustrates the channel topology of the ATS structure and the adsorption location of IPA (i.e., green spheres).

The free energy analysis is conducted to better understand the unusually low permeation flux observed for ATS as noted above. Interestingly, the slow permeation is found to be attributed to the presence of many moderate barriers with each being as high as ∼5 *k*_B_*T* (∼3.3 kcal/mol, Figure 5(a)). To further shed light on this aspect, the intermolecular interaction profile, including both van der Waals and Coulombic forces, is further quantified. Figure 5(b) clearly shows that IPA molecules experience very stable interaction energies of as low as −25 kcal/mol, corresponding to the corner of zigzag channels within the bulk membrane. IPA molecules are essentially sort of being trapped in those deep wells, leading to a large barrier and thus a slow permeation. While zeolites with a strong affinity toward IPA may be typically deemed promising, their topologies might hinder the permeation of IPA and correspondingly significantly reduce flux and selectivity.

The inset shown in (b) illustrates the channel topology of the ATS structure and the adsorption location of IPA (i.e., green spheres).

### Effect of the Feed-Side Surface

**Figure 6:**
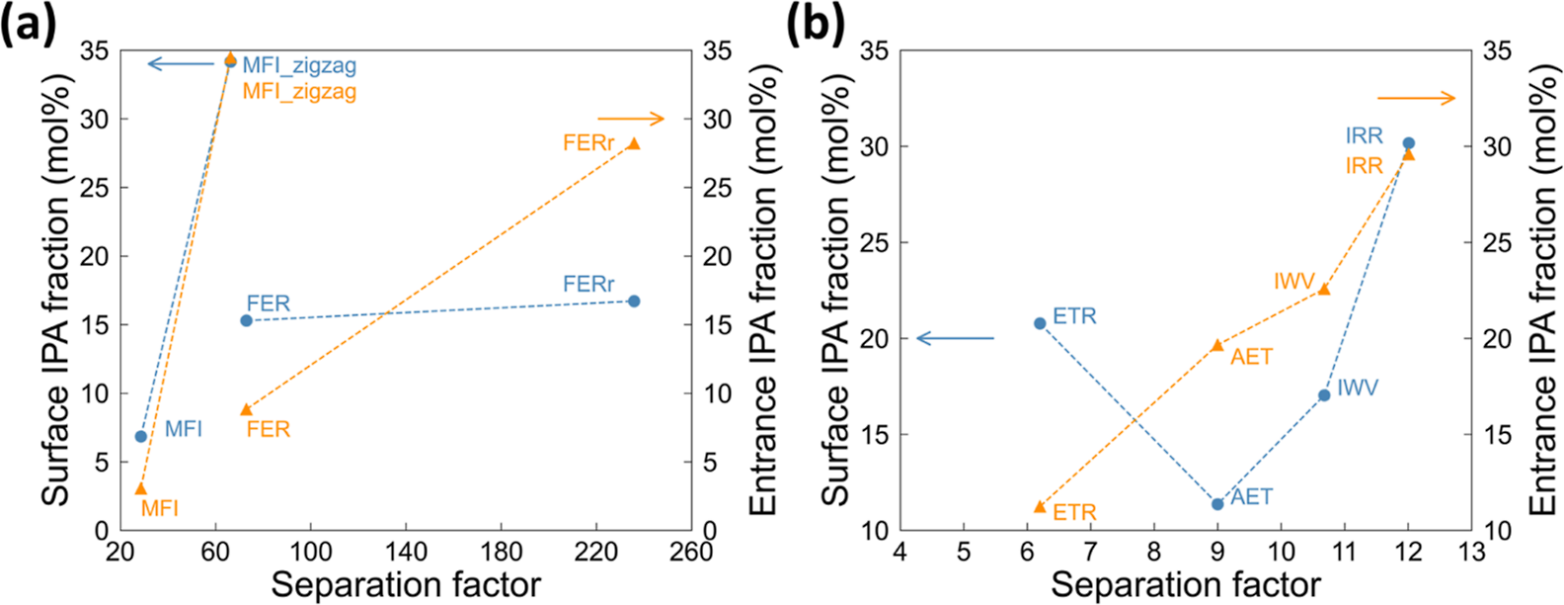
Surface (left-axis) or entrance (right-axis) IPA fraction versus the separation factor for two groups of nanosheets: (a) MFI, MFI_zigzag, FER, and FERr, and (b) AET, IWV, ETR, and IRR. Structures in each group have a similar or essentially identical IPA concentration in their bulk structure.

Aside from the above-discussed adsorption selectivity of the bulk structure, it is evident that other factors also influence the overall IPA/water separation performance. As shown in Figure 3(a), the adsorption selectivity cannot fully explain the observed separation selectivity. For instance, while FERr exhibits an IPA mole fraction of 0.91 in the bulk membrane that is nearly identical to FER, the former demonstrates a notably greater separation factor (i.e., 235.86 vs 72.93, respectively). Despite AET also having a favorable IPA mole fraction of 0.86, it only offers a rather low separation factor of 9. Considering the ultrathin-film nature of nanosheet membranes, their surface characteristics should obviously be taken into account. Specifically, as would be intuitively expected, the first step of the separation process starts from the surface adsorption of the mixture onto the feed-side surface. To this end, this study further quantified the IPA mole fraction on the feed-side surface and also particularly near the channel entrances per molecular trajectories (see Figure S1 for more details). Specifically, to better probe the effect of feed-side surfaces, only those with similar bulk membrane selectivity are discussed. As shown in Figure 6(a), MFI_zigzag and FERr exhibit a higher surface IPA fraction compared to MFI and FER, respectively. As a result, the separation factors of MFI_zigzag and FERr (66.27 and 235.86) both indeed outperform MFI and FER (28.64 and 72.93). Moreover, our results also indicate that the entrance IPA fraction is more representative than the surface IPA fraction for its direct relevance. While FERr exhibits a separation factor three times greater than FER, the former only has a marginally higher surface concentration by 1.4 mol %. By contrast, the entrance IPA concentration of FERr is instead notably higher by 20 mol %. A similar observation can also be seen in Figure 6(b) for another group of nanosheets of a greater pore size. The entrance IPA concentration again demonstrates a stronger correlation with the separation factor, compared to the nonproportional relationship between the surface IPA concentration and the separation factor. It is also found that the adsorption characteristics on the feed side are greatly affected by the number of silanol groups on the surface. Owing to the intrinsic hydrophilicity of silanol groups, structures showing a stronger hydrophobicity (e.g., MFI_zigzag) generally have a fewer silanol number.

### Effect of the Permeate-Side Surface

**Figure 7:**
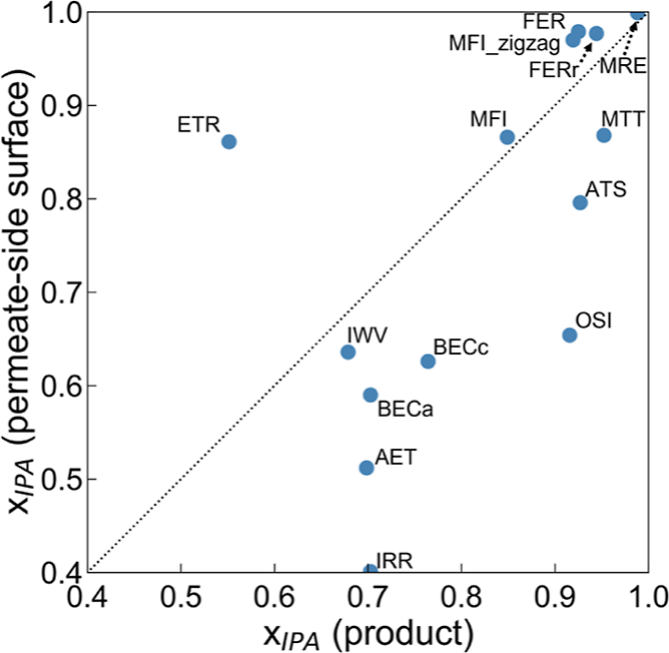
Relationship between the IPA mole fraction (*x*_IPA_) on the permeate side surface and that of the product collected on the adsorbing plate.

This study has also found the profound role of the permeate-side surface, which has been greatly overlooked in the literature. As shown in Figure 7, a surprisingly notable variation between the IPA concentration in the liquid film formed on the permeate-side surface and that of the product collected on the adsorbing plate is found, suggesting that the evaporation process on the permeate-side surface may be strongly controlled by its characteristics. Overall, the majority of zeolite candidates show an enriched IPA concentration after evaporation. This is reasonable, provided that the vapor pressure of IPA is greater than that of water. However, the extent of such enrichment is interestingly found to dramatically vary. For example, the IPA product concentration with the OSI nanosheet is enhanced to 0.916 from 0.654 on the surface, while it only increases from 0.636 to 0.679 for IWV. Moreover, the results show that the higher vapor pressure of IPA in fact does not necessarily lead to an enriched IPA concentration; the IPA concentration after evaporation can even decrease (i.e., from 0.861 to 0.551 for ETR).

**Figure 8:**
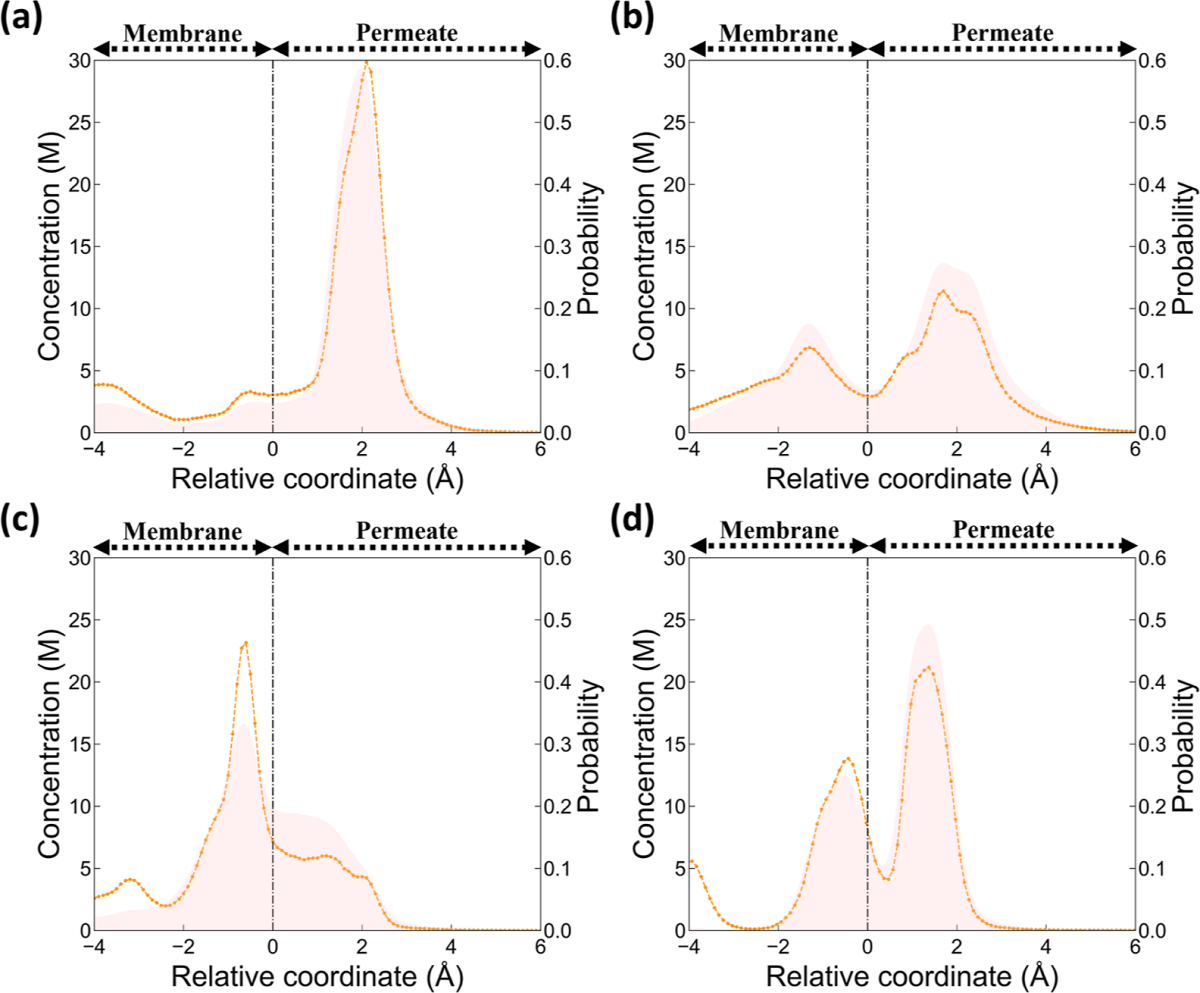
Density profiles (dotted lines) of IPA molecules and the spatial distribution (pink-shaded areas) of the location of IPA molecules before evaporating from the surface for (a) OSI, (b) IWV, (c) ETR, and (d) FERr. The black dotted lines represent permeate-side surfaces of zeolites.

To explore the effect of the permeate-side surface, four zeolite nanosheets (i.e., OSI, IWV, ETR, FERr) are investigated in detail. Table 2 summarizes their separation factor, flux, and the IPA concentration on both the permeate-side surface and the adsorbing plate. Specifically, the density profiles of IPA molecules adsorbed on their permeate-side surface are analyzed to inform their preferable location on the surface. Moreover, the spatial distribution on the permeate-side surface for those IPA molecules before evaporating from the surface is also quantified. It is found that the observed variation in the above-mentioned enrichment effect is largely affected by the IPA distribution. Comparing OSI and IWV, while both structures have a nearly identical permeate-side molar fraction (0.654 and 0.636, respectively), they show largely different collected IPA concentrations (0.916 and 0.679, respectively). Distinctly, Figure 8(a) demonstrates that the former exhibits a pronounced IPA peak in the surface liquid film, thus facilitating the likelihood of IPA evaporation. By contrast, IWV possesses a notably lower concentration level on the surface and moreover, two distinctive peaks are present. Such two peaks may lead to a prolonged retention time for IPA molecules, as they may experience back-and-forth motion between the two peaks as can be observed in Figure S3, which makes the evaporation of IPA from the surface less effective. Specifically, such behavior, denoted as diffusion-back motion, was also identified very recently by Wang et al.^51^ Another intriguing case is the much reduced IPA concentration after evaporation (i.e., 0.861 to 0.551) for the ETR nanosheet. Figure 8(c) reveals that an IPA-concentrated liquid film on the permeate-side surface is absent (i.e., only ∼6 M between positions 0 and 2 Å) and moreover, there is interestingly a strong IPA peak located inside the membrane. The former results in a weak driving force for IPA evaporation, while the strong IPA adsorption well may trap IPA to slow down evaporation. Indeed, for the latter, Figure 8(c) shows that there is a discrepancy between the density profile of IPA and the spatial distribution of IPA molecules before evaporating from the surface. Those IPA molecules before evaporating from the surface, compared to the overall density profile, appear to be less likely to be located at the strong peak inside the membrane. As for FERr, while it also has a prominent IPA peak on the surface (Figure 8(d)), two pronounced peaks do present and thus lead to the less desirable diffusion-back motion as can also be seen in Figure S3. As such, FERr ends up to have a slightly lower purity after evaporation (i.e., 0.977 to 0.944).

**Table 2:** Separation Factor, Flux, and IPA Concentration (*x*_IPA_) on the Permeate-Side Surface and on the Adsorbing Plate for OSI, IWV, ETR, and FERr

structure	separation factor	flux (kg/m^2^ hr)	*x*_IPA_ (permeate-side surface)	*x*_IPA_ (product)
OSI	55.07	44031.6	0.654	0.916
IWV	10.68	51923.3	0.636	0.679
ETR	6.20	18256.2	0.861	0.551
FERr	235.86	26608.7	0.977	0.944

**Figure 9:**
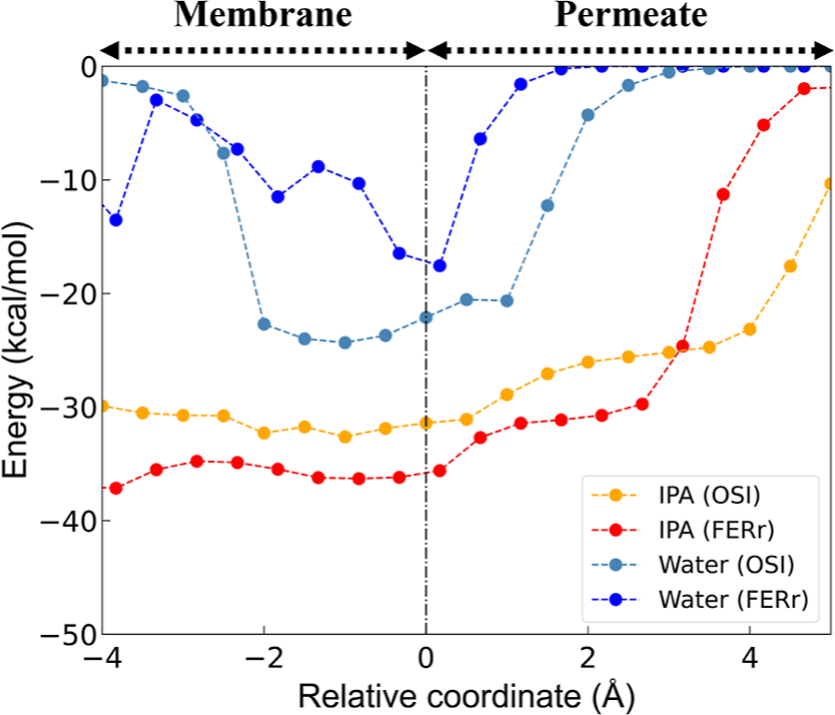
Energy profile of IPA and water molecules near the permeate-side surface along the permeation direction for OSI and FERr membranes. The black dotted line indicates permeate-side surfaces of zeolites.

From the above discussion, the existence, number, and location of prominent IPA concentration peaks near the permeate-side surface appear to affect the enrichment of IPA after evaporation. We find that the potential energy level of IPA molecules also plays a critical role. By comparing the above-discussed OSI and FERr, the former exhibits the most significant enrichment after evaporation while the latter has a slightly reduced IPA product concentration. Although FERr suffers from diffusion-back motion to a certain extent, both of the membranes have a very pronounced IPA peak in the liquid film formed on the permeate-side surface. Interestingly, Figure 9 shows that IPA molecules on the permeate-side surface of OSI experience a higher (i.e., weaker) interaction level of −30 ∼ −25 kcal/mol as compared to that of FERr (i.e., −35 ∼ −30 kcal/mol). Besides, the adsorption energy of water molecules also shown in Figure 9, is found critical as well. Water molecules in OSI experience a relatively more favorable interaction of ∼ (−24 kcal/mol), compared to that of > −20 kcal/mol in FERr, near the surface (i.e., between positions −2 and 1 Å). Their permeation may therefore be hindered, promoting the enrichment effect.

## Conclusions

Through molecular simulations, this study systematically investigates the potential of zeolite nanosheets as pervaporation (PV) membranes in IPA/water separation. The results demonstrate the great promise of zeolite nanosheets, offering an impressive IPA-to-water separation factor of more than 400. This can extract nearly anhydrous IPA of more than 99 wt % from a 40 wt % IPA mixture with merely a single step. The separation factor of nanosheets is found to be predominantly controlled by the adsorption selectivity of IPA over water in the bulk membrane. From the free energy perspective, a highly selective IPA membrane essentially poses a significant permeation barrier for water to enter the membrane. However, in certain cases, a substantial energy barrier may exist for IPA in the bulk structure, thus hindering its diffusion. Furthermore, structures having an LCD of around 6 Å are identified to likely offer a higher IPA-to-water separation factor. The study has also shown that the PV performance of zeolite nanosheets is influenced by their external surfaces. It is preferable to have a more hydrophobic feed-side surface, particularly near the channel entrance, while having a single prominent IPA peak in the permeate-side liquid film. In summary, this study showcases the considerable potential of zeolite nanosheets as pervaporation membranes for IPA/water separation and offer insights into the design of ultrathin-film nanoporous PV membranes. Validation of the results observed in this study may be to be conducted by experiments. Though, this may associate with some notable challenges such as the complexity of synthesizing ultrathin and pure silica zeolite nanosheets with minimized defects. Provided that the IPA-to-water PV performance is largely controlled by the adsorption selectivity in the bulk materials, experimentally validating the adsorption properties of materials of interest may be an important first step, before making significant efforts into their synthesis of ultrathin film nanosheet membranes.

## Supporting Information Available

Additional tables and figures referred to in the main text, which include geometric features and MD-predicted performance of all studied nanosheet structures, illustration of different membrane regions, density profiles of IPA and/or water, and the spatial distribution of the location for IPA molecules on the permeate-side surface before evaporation and the molecular trajectories of some selected IPA molecules (PDF)
