# Supplementary material for: Pervaporation Separation of Isopropanol/Water Using Zeolite Nanosheets: A Molecular Simulation Study
Source: J Phys Chem B. 2024 Aug 26;128(35):8546–56. doi: 10.1021/acs.jpcb.4c04237 (PMC11382281; doi:10.1021/acs.jpcb.4c04237)
Supplement: Supplementary file 1 — jp4c04237_si_001.pdf [file jp4c04237_si_001.pdf]

Supplementary Information

for

Pervaporation Separation of Isopropanol/Water Using Zeolite Nanosheets: A Molecular Simulation Study

Ming-Yen Tsai[a], *Li-Chiang Lin*[a,b]*

[a]Department of Chemical Engineering, National Taiwan University, No. 1, Sec. 4, Roosevelt Road, Taipei 10617, Taiwan

[b]William G. Lowrie Department of Chemical and Biomolecular Engineering, The Ohio State University, 151 W. Woodruff Avenue, Columbus, OH 43210, United States.

*Email: lclin@ntu.edu.tw

1. Tables referred to in the main text

Table S1 Structural properties of zeolite nanosheets investigated in this study.

Structure	*PLD (Å)[a]*	*LCD (Å)[a]*	*Accessible volume (%)[a]*	*Channel dimensionality[b]*	*Channel density (nm^{-2})[c]*
**FERr**	4.29	5.42	8.40	1	0.74
**FER**	4.29	5.42	8.40	1	0.74
**MFI**	4.30	5.94	8.80	1	0.76
**MFI_zigzag**	4.30	5.94	8.80	1	0.76
**MTT**	4.56	5.55	6.74	1	0.80
**MRE**	5.59	6.36	5.74	1	0.68
**OSI**	5.88	6.26	8.64	1	0.58
**BECa**	5.91	6.24	19.01	3	0.60
**BECc**	5.91	6.24	19.01	3	0.60
**ATS**	6.37	6.58	14.23	1	0.70
**IWV**	6.63	8.14	20.73	2	0.55
**AET**	7.17	7.77	12.38	1	0.42
**ETR**	8.93	9.61	17.78	1	0.27
**IRR**	11.71	13.92	36.49	3	0.32

[a]The pore limiting diameter (PLD), the largest cavity diameter (LCD), and accessible volume (%) are calculated by Zeo++.[1,2] [b]Channel dimensionality is determined by whether the zeolite has channels in crystallographic a-, b-, or c-directions, allowing solution molecules (i.e., water and IPA) to diffuse along these directions. [c]Channel density is the number of channels along the permeation direction divided by the cross-sectional area of the membrane.

Table S2 Separation factor, flux, and the IPA mole fraction (x_{IPA}) at different regions of studied nanosheet membranes.

Structure	*Separation factor*	*Flux (kg/m² hr)*	x_{IPA} *(bulk membrane)*	x_{IPA} *(permeate-side surface)*	x_{IPA} *(product)*
**FERr**	235.86	26608.7	0.913	0.977	0.944
**FER**	72.93	19818.4	0.921	0.979	0.925
**MFI**	28.64	9956.9	0.816	0.866	0.849
**MFI_zigzag**	66.27	27649.9	0.985	0.970	0.919
**MTT**	100.47	20201.3	0.936	0.868	0.952
**MRE**	431.92	41504.9	0.991	0.999	0.988
**OSI**	55.07	44031.6	0.935	0.654	0.916
**BECa**	14.91	22062.3	0.884	0.590	0.703
**BECc**	16.39	49497.5	0.967	0.626	0.764
**ATS**	35.56	13702.8	0.947	0.796	0.927
**IWV**	10.68	51923.3	0.809	0.636	0.679
**AET**	9.00	69251.9	0.863	0.512	0.698
**ETR**	6.20	18256.2	0.757	0.861	0.551
**IRR**	12.01	66796.5	0.692	0.401	0.703

2. Figures referred to in the main text

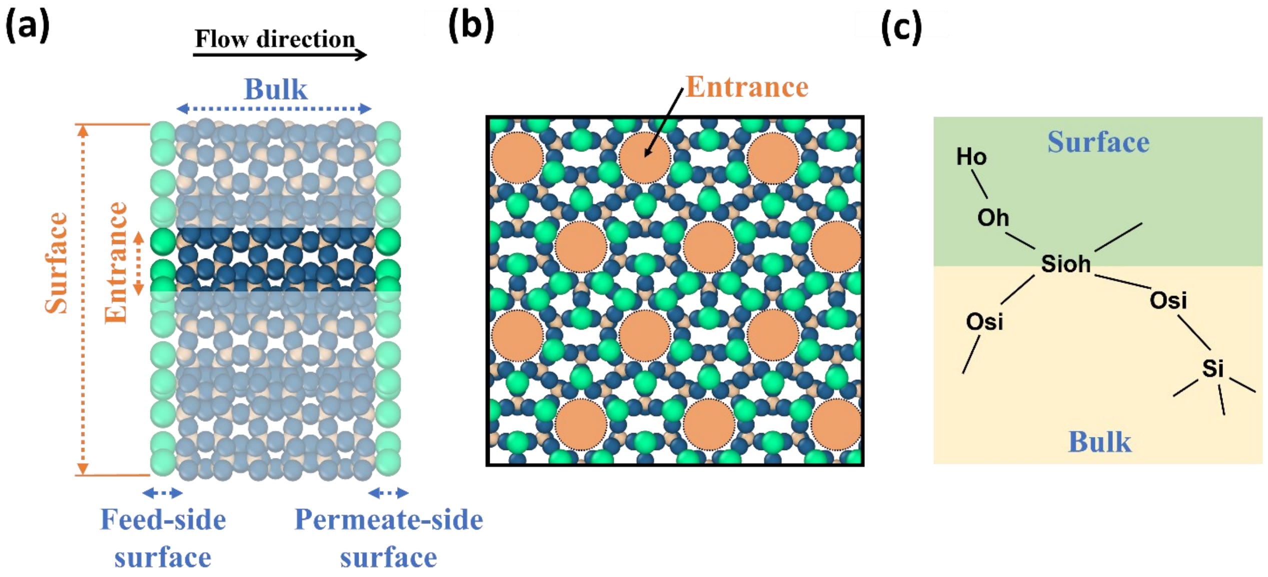

Figure S1 The (a) side view and the (b) top view of different membrane regions (i.e., surface, entrance, and bulk) defined in this work. The thickness of the feed-side surface and permeate-side surface is 5 Å measured from the furthest silicon atom on the surface (i.e., denoted as Sioh as shown in (c)), while the entrance region is similar to the surface region but only includes the channel entrances (represented by the orange circles in (b)). The bulk is the region between Sioh at the feed-side surface and that at the permeate-side surface. In (a-b), the silanol group, Si, and O are represented by green, bronze, and blue colors, respectively. In (c), Ho, Oh, Sioh, Osi, and Si respectively refer to the hydrogen, oxygen, silicon connected to silanol groups, oxygen in the bulk region, and silicon in the bulk region.

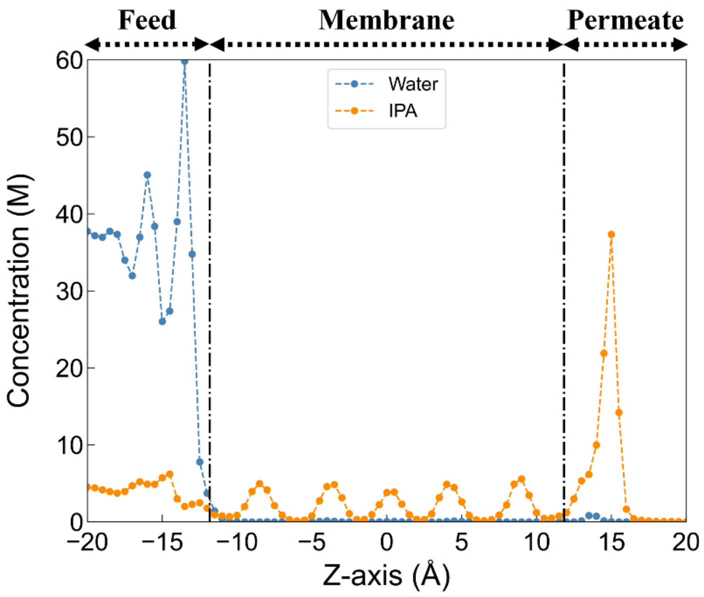

Figure S2 Concentration profiles of water and IPA across the MRE nanosheet membrane with the black dotted lines indicating the locations of its external surfaces.

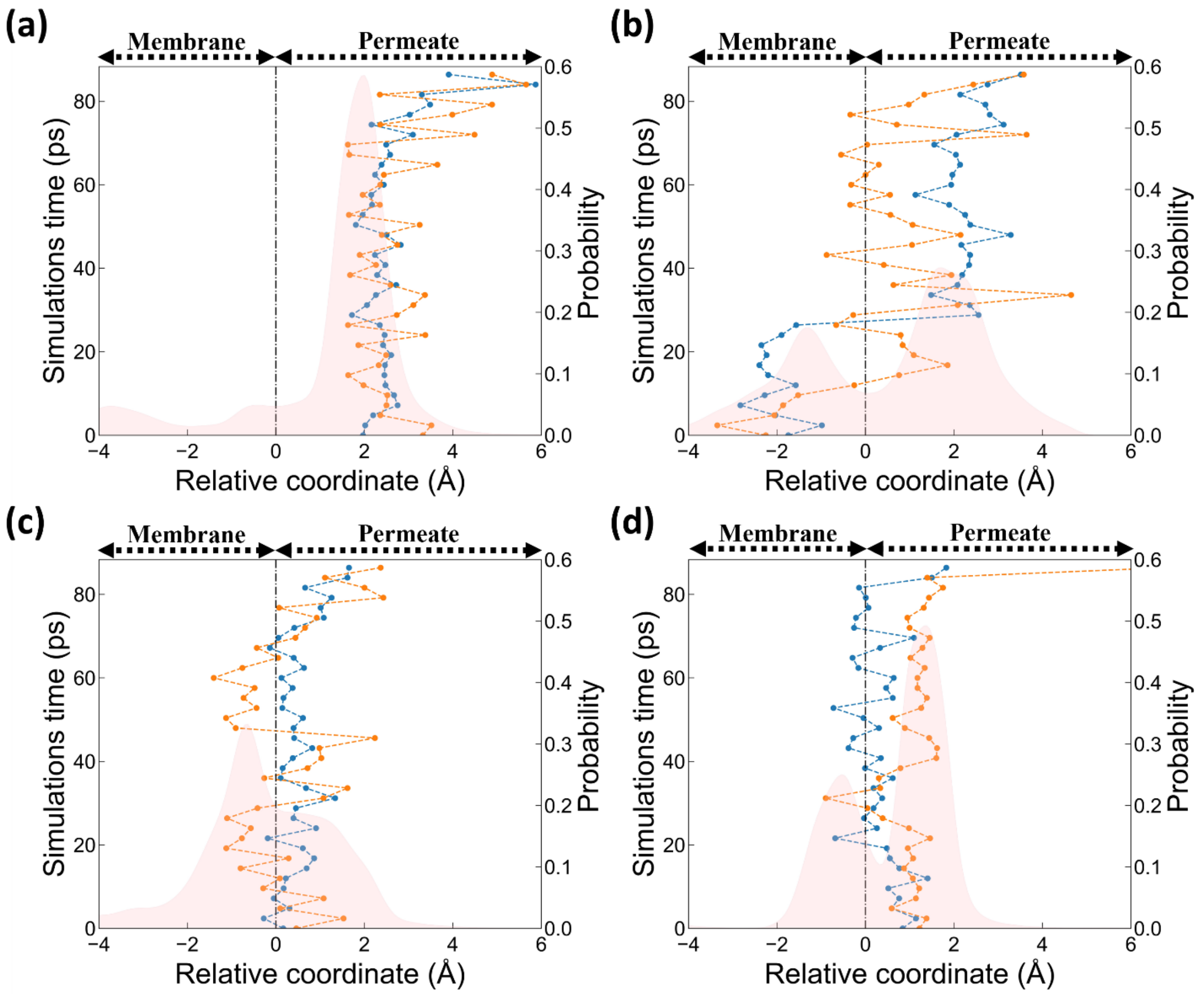

Figure S3 The spatial distribution (pink-shaded areas) of the location of those IPA molecules before they evaporate from the permeate-side surface (black dotted line) for (a) OSI, (b) IWV, (c) ETR, and (d) FERr. The molecular trajectories of two selected IPA molecules before evaporating from the surface are also shown by blue and orange dotted lines.

3. References

(1) Willems, T. F.; Rycroft, C. H.; Kazi, M.; Meza, J. C.; Haranczyk, M. Algorithms and tools for high-throughput geometry-based analysis of crystalline porous materials. *Microporous Mesoporous Mater.* **2012**, *149* (1), 134-141.

(2) Martin, R. L.; Smit, B.; Haranczyk, M. Addressing Challenges of Identifying Geometrically Diverse Sets of Crystalline Porous Materials. *J. Chem. Inf. Model.* **2012**, *52* (2), 308-318.
